# Ethylene polymerization using *N*-Heterocyclic carbene complexes of silver and aluminum

**DOI:** 10.1080/15685551.2023.2229641

**Published:** 2023-07-05

**Authors:** Nanako Kimura, Daisuke Takeuchi, Sayoko Ogura, Ayaka Takazawa, Masaki Kakiage, Takeshi Yamanobe, Hiroki Uehara

**Affiliations:** a Graduate School of Science & Technology, Hirosaki University, Hirosaki, Aomori, Japan; b Graduate School of Science & Technology, Gunma University, Kiryu, Gunma, Japan

**Keywords:** Silver, aluminum, N-heterocyclic carbene, ethylene polymerization, ultra-high molecular weight polyethylene

## Abstract

Various transition metal catalysts have been utilized for ethylene polymerization. Silver catalysts have attracted less attention as the catalysts, but are potential for production of high molecular weight polyethylene. Herein, we report that silver complexes with various *N*-heterocyclic carbene (NHC) ligands in combination with modified methylaluminoxane (MMAO) afford polyethylene with high molecular weight (melting point over 140°C). SEM observation showed that the produced polyethylene has ultra-high molecular weight. NMR investigation of the reaction between the silver complexes with organoaluminums indicate that the NHC ligands transfer from the silver complex to aluminum to produce NHC aluminum complexes. Ph_3_C[B(C_6_F_5_)_4_] abstract methyl group from the NHC aluminum complex to afford cationic aluminum complex. The NHC aluminum complex promoted ethylene polymerization in the presence of Ph_3_C[B(C_6_F_5_)_4_] and organoaluminums. NHC ligand also promoted ethylene polymerization in combination with MMAO to produce polyethylene with high melting point (140.7°C). Thus, the aluminum complexes are considered to be the actual active species in silver-catalyzed ethylene polymerization.

## Introduction

Transition metal catalysts have been extensively used for ethylene polymerization since the discovery of Ziegler-Natta catalyst. There has been extensive study on Ziegler-Natta type heterogeneous catalyst [1–3]. In addition, various homogeneous catalysts have been reported to promote the polymerization. Group 4 metals (Ti, Zr, Hf), rare earth metals, V, Cr, Fe, Co, Ni and Pd are main players in this field [4]. Other examples of polymerization catalysts of minor transition metals, such as Mn [5–9], Nb [10–15], Ta [12,16–18], Ru [19–23], Rh [22,24–28], Ir [22], and Pt [28] have been also known. Copper complexes have been also known to promote ethylene polymerization [29–31], but detailed studies indicate that aluminum complex, which is formed by ligand transfer from copper complexes to organoaluminum cocatalyst, is the actual active species in some cases [32].

**Scheme 1.**
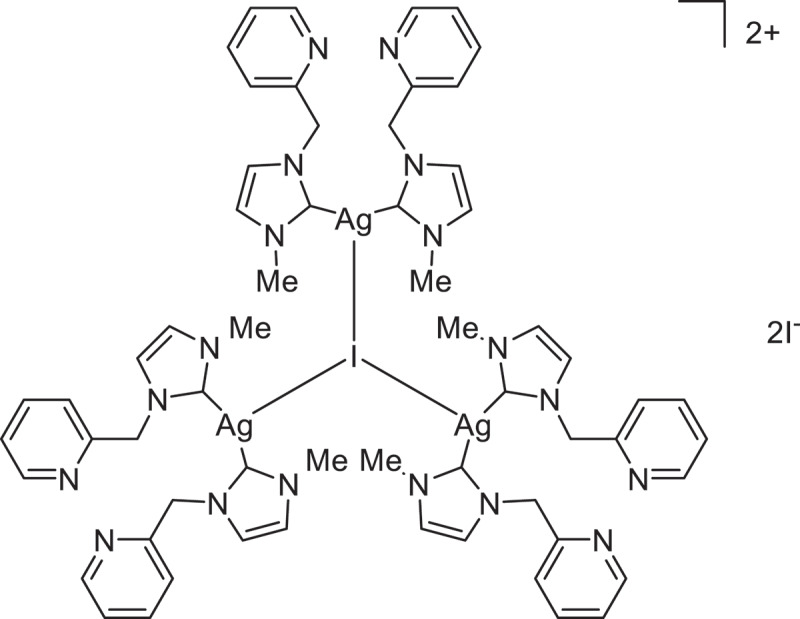
Trinulcear silver complex catalyst for ethylene polymerization [33].

Silver is another minor transition metal that is known to promote ethylene polymerization. Although active species of most of the above-mentioned olefin polymerization catalysts are cationic alkyl species, such species is not common in silver complex, as monovalent species is common in silver. Silver complexes are often light sensitive and need care in handling. These would be the possible reason why silver catalysts have attracted less attention as olefin polymerization catalysts. However, Jin reported that trinuclear silver complex with pyridyl-substituted *N*-heterocyclic carbene (NHC) ligand shows high activity for ethylene polymerization in the presence of methylaluminoxane (MAO) (Scheme 1) [33]. The produced polyethylene is rarely soluble in common organic solvents, and no further characterization of the produced polyethylene has been demonstrated. The detailed mechanism of the polymerization, such as active species of the polymerization, is not clear, either. In the present paper, we examined ethylene polymerization by silver as well as copper and gold complexes with various NHC ligand in the presence of MAO.

## Results and discussion

### Ethylene polymerization by NHC silver/organoaluminum catalyst systems

**Figure 1.**
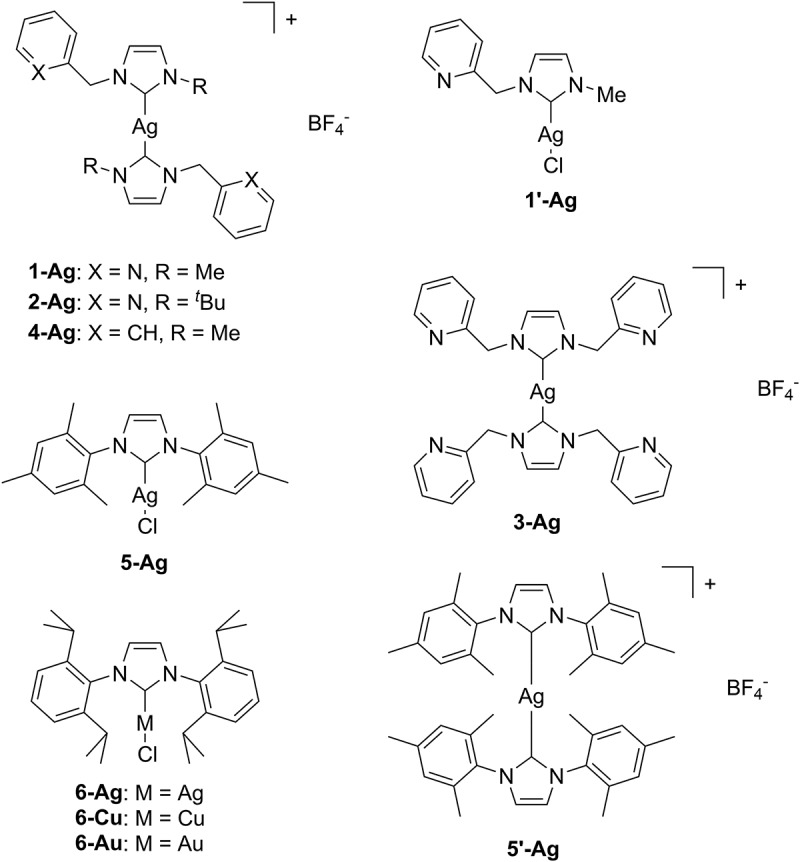
Ag, Cu, and Au complexes used in this study.

Ethylene polymerization was examined using various NHC silver complexes in combination with organoaluminum cocatalyst (Table 1). Figure 1 shows the NHC silver complexes used in this study. First, we attempted to synthesize trinuclear silver complex with 1-methyl-3-(pyridylmethyl)imidazolylidene ligand reported by Jin [33], but it was not successful. Thus, we synthesized some mononuclear silver complexes with various NHC ligands, including 1-methyl-3-(pyridylmethyl)imidazolylidene ligand, and examined them for ethylene polymerization. Silver complex with 1-methyl-3-(pyridylmethyl)imidazolylidene ligand (**1-Ag**) brought about polymerization in the presence of modified methylaluminoxane (MMAO) [34]. The activity was 0.103 g mmol_Ag_^−1^ h^−1^ (Table 1, run 1), which is much lower than that by trinuclear silver complex reported by Jin (470 g mmol_Ag_^−1^ h^−1^) [33]. Silver complexes with 1-*tert*-butyl-3-(pyridylmethyl)imidazolylidene ligand (**2-Ag**) and 1,3-bis(pyridylmethyl)imidazolylidene ligand (**3-Ag**) were also effective for the polymerization (runs 2 and 3), although their activity was lower than **1-Ag** with *N*-methyl group (run 1). Silver complex with 1-methyl-3-benzyl-imidazolylidene ligand (**4-Ag**), without pyridyl group, also produced the polymer in lower catalytic activity (run 4). These results indicate the importance of pyridyl group in the NHC ligand in the ethylene polymerization. The commercially available NHC silver complexes with IMes (1,3-bis(2,4,6-trimethylphenyl)imidazol-2-ylidene) (**5-Ag**) or IPr (1,3-bis(2,6-diisopropylphenyl)imidazol-2-ylidene) (**6-Ag**) ligands also catalyzed the polymerization (runs 5 and 6), but in lower catalytic activity compared to those with pyridyl group on the NHC ligand (runs 1–3).

**Table 1.** Ethylene polymerization by Ag, Cu, and Au complexes with NHC ligand/organoaluminum catalyst systems^.a^

	NHC complex		Al						
run		/mmol		/mmol	yield /g	activity /g mmol _metal_^−1^ h^−1^	*M*_w_	*M*_w_*/M*_n_	*T*_m_^*b*^ /°C
1	**1-Ag**	0.020	MMAO	31	0.0412	0.103	- ^*d*^	- ^*d*^	139.2
2	**2-Ag**	0.020	MMAO	31	0.0195	0.0488	- ^*d*^	- ^*d*^	142.6
3	**3-Ag**	0.020	MMAO	31	0.0254	0.0635	- ^*d*^	- ^*d*^	-^*d*^
4	**4-Ag**	0.020	MMAO	31	0.0041	0.0103	- ^*d*^	- ^*d*^	- ^*d*^
5	**5-Ag**	0.020	MMAO	31	0.0079	0.0198	- ^*d*^	- ^*d*^	- ^*d*^
6	**6-Ag**	0.020	MMAO	31	0.0124	0.0310	2,670,000	14.7	140.9
7	**5’-Ag**	0.010	MMAO	31	0.0091	0.023	- ^*d*^	- ^*d*^	138.3
8^*c*^	**1-Ag**	0.010	MMAO-12	31	0.0234	0.090	- ^*d*^	- ^*d*^	141.7
9^*c*^	**1-Ag**	0.010	MMAO	31	0.0234	0.090	- ^*d*^	- ^*d*^	141.4
10	**1-Ag**	0.020	MMAO	0.3	0.0015	0.0038	- ^*d*^	- ^*d*^	- ^*d*^
11	**1-Ag**	0.010	MAO	31	0.0399	0.200	- ^*d*^	- ^*d*^	- ^*d*^
12^*d*^	**1-Ag**	0.010	MAO	10	0.0180	0.0750	- ^*d*^	- ^*d*^	143.7
13^*e*^	**1-Ag**	0.010	MAO	15.5	0.0048	0.0120	- ^*d*^	- ^*d*^	- ^*d*^
14^*f*^	**1-Ag**	0.010	MAO	31	0.0201	0.0503	- ^*d*^	- ^*d*^	- ^*d*^
15	**2-Ag**	0.020	DEAC	31	0.0024	0.0060	- ^*d*^	- ^*d*^	129.3
16^*g*^	**2-Ag**	0.020	MMAO	31	0.0214	0.0535	1,410,000	13.2	138.4
17	**6-Cu**	0.020	MMAO	31	0.0174	0.0435	329,000	1.57	141.3
18	**6-Au**	0.020	MMAO	31	0.0064	0.0160	15,600,000	60.2	140.0

^*a*^Reaction Conditions: toluene = 20 mL, temp. = 30°C, ethylene = 1MPa, 20 h, unless otherwise noted. ^*b*^Determined by DSC, 1st scan. ^*c*^time = 26 h. ^*d*^time = 24 h. ^*e*^toluene = 10 mL. ^*f*^ethylene = 2 MPa. ^*g*^Aging at 30°C for 18 h before starting polymerization. ^*h*^Not determined.

Bis(NHC) silver complex, [(IMes)_2_Ag]BF_4_ (**5’-Ag**) (run 7) showed similar catalytic activity as mono(NHC) complex (**5-Ag**) (run 5). Jin reported that the polyethylene obtained by the trinuclear silver complex/MAO is insoluble in common organic solvents. Most of the polyethylenes obtained by the mononuclear silver complexes in this study were also sparingly soluble in common organic solvents, such as 1,2-dichlorobenzene and 1,2,4-trichlorobenzene. Gel permeation chromatography (GPC) analysis of the 1,2,4-trichlorobenzene-soluble part of the polyethylene obtained by **6**-**Ag**/MMAO showed that *M*_w_ is over 10^6^ (run 6).

**Figure 2.**
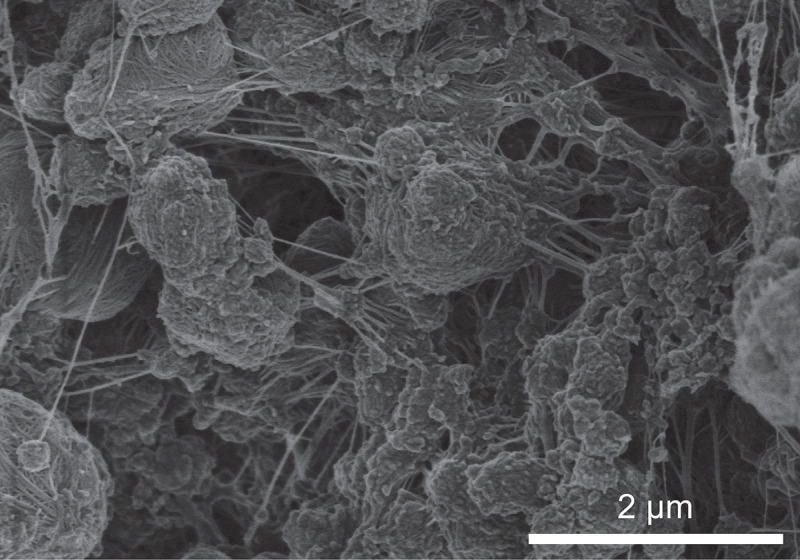
SEM image of polyethylene obtained by silver catalyst (Table 1, run 8).

Differential scanning calorimetry (DSC) analysis of the polyethylene obtained by the silver complexes show their melting point at 138–143°C (1st heating), which are characteristic of ultra-high molecular weight polyethylene with highly linear structure [35]. Thus, the low solubility of the produced polyethylene would be because they are ultra-high molecular weight. MMAO-12, which has octyl-Al bond, was also effective as cocatalyst, and afforded polyethylene with *T*_m_ = 141.4°C (run 8). Cob-web morphology was observed in scanning electron microscope (SEM) image of the obtained polyethylene (Figure 2), which is characteristic of polyethylene powders as polymerized [36]. Decrease in concentration of the silver complex (**1-Ag**) resulted in almost comparable catalytic activity (runs 9 and 1) and formation of polyethylene with *T*_m_ = 141.4°C. However, decrease in concentration of MMAO lead to significant decrease in catalytic activity (runs 10 and 1).

MAO in combination with **1-Ag** produced polyethylene with higher catalytic activity to **1-Ag**/MAO (runs 11 and 9). In this case, again decrease in catalytic activity was observed when the concentration of MAO was decreased (runs 12, 13, and 11). Increase in ethylene pressure has negative effect on catalytic activity (run 14). **2-Ag**/diethylaluminum chloride (DEAC) showed much lower activity than **2-Ag**/MMAO, and afforded polyethylene with lower melting point (runs 15). The actual catalytic species in the present silver/organoaluminum catalyst systems is considered to be a cationic aluminum complex, formed by the ligand transfer from silver to aluminum (*vide infra*). The lower Lewis acidic character of DEAC compared to MAO and/or the presence of chloride on the cationic aluminum species would be the possible reason for the low activity. In order to promote efficient ligand transfer from silver to aluminum before the polymerization, aging the catalyst was conducted at 30°C for 18 h before starting polymerization. However, it showed small positive effect on the catalyst activity (run 16 and 2).

In addition to NHC silver complexes, NHC copper and NHC gold complexes (**6-Cu** and **6-Au**) were also examined for the ethylene polymerization (runs 13 and 14). Actually, both copper and gold complexes were effective for the ethylene polymerization in the presence of MMAO. The copper complex showed slightly higher catalytic activity than the corresponding silver complex, whereas the activity of the corresponding gold complex was much lower. The melting points of the produced polyethylenes obtained by Cu, Ag, and Au complexes, however, were above 139°C, and formation of ultra-high molecular weight polyethylene was indicated in all these cases.

Several copper complexes have been reported to promote ethylene polymerization in the presence of organoaluminums [29–31]. In these cases, however, the actual active species is proposed to be organoaluminum complex formed via ligand transfer from the copper complex to the organoaluminum [32]. There has been no report on gold catalyst capable of promoting ethylene polymerization. Thus, it is anticipated that the active species in the present polymerization is NHC organoaluminum complex, formed via transfer of NHC ligand from the silver, copper, or gold complex to aluminum complex, rather than the organosilver, organocopper, or organogold complex.

## NHC ligand transfer from silver to aluminum

**Figure 3.**
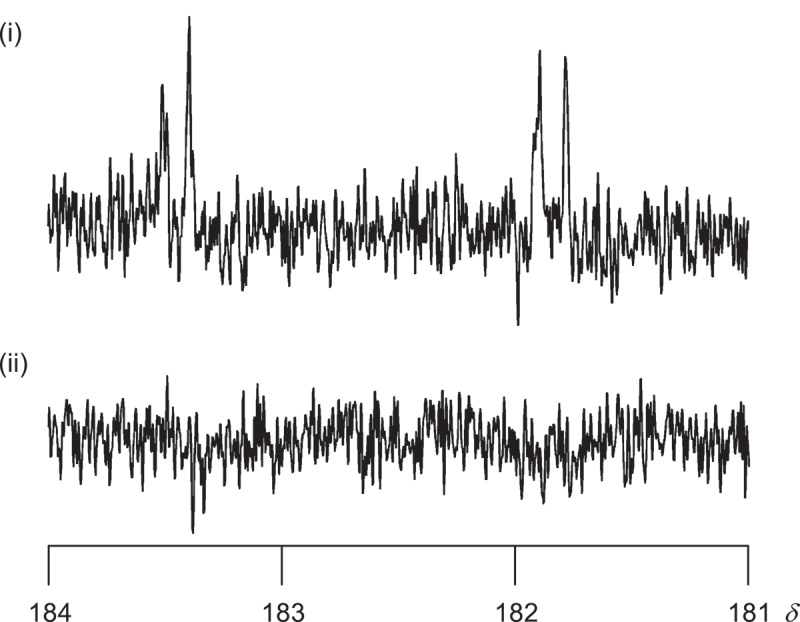
^13^C{^1^H} NMR spectra of the reaction mixture of **6-Ag** and ^i^Bu_3_Al in C_6_D_6_ measured at (i) 3 h and (ii) 24 h after the preparation of the reaction mixture.

In order to confirm that the NHC aluminum complex actually forms in the present polymerization, the NMR study of the reaction between NHC silver complex and organoaluminum was conducted. Thus, (IPr)AgCl (**6-Ag**) (0.12 mmol) and ^i^Bu_3_Al (0.12 mmol) were mixed in C_6_D_6_ (0.5 mL) and reacted at room temperature. ^13^C{^1^H} NMR of the mixture (3 h after the preparation of the mixture) showed doublet of doublet signal due to quaternary carbon of the IPr ligand bonded to Ag at δ 182.6 (J_13C-107Ag_ = 188.4 Hz, J_13C-109Ag_ = 214.8 Hz) (Figure 3 (i)), whereas the signal was absent after 24 h (Figure 3 (ii)). This result indicates the dissociation of the IPr ligand from the silver center.

It is anticipated that the IPr ligand, dissociated from the silver center, is transferred to aluminum. ^1^H NMR spectrum of the mixture after 24 h showed a new doublet signal at δ −0.33, which is assigned to AlC*H*_2_CH(CH_3_)_2_ attached to IPr (Figure S1). The spectrum also showed a singlet signal at δ 6.65 as well as triplet signals at δ 7.39, 7.21, and 7.03 with intensity ratio of 1.00 : 0.88 : 3.96. This result indicates that (IPr)AliBu_3_ complex, once formed via the ligand transfer from Ag to Al, is transformed to zwitter ionic species. It has been reported that the reaction of IPr with R_3_Al affords (IPr)AlR_3_, which is easily transformed to zwitterionic species due to the steric repulsion between bulky NHC ligand with alkyl group on aluminum center [37,38]. The similar reaction also took place in the present reaction. The slow ligand transfer reaction from Ag to Al would be also due to the steric bulkiness of the ^i^Bu_3_Al and IPr ligand.

**Figure 4.**
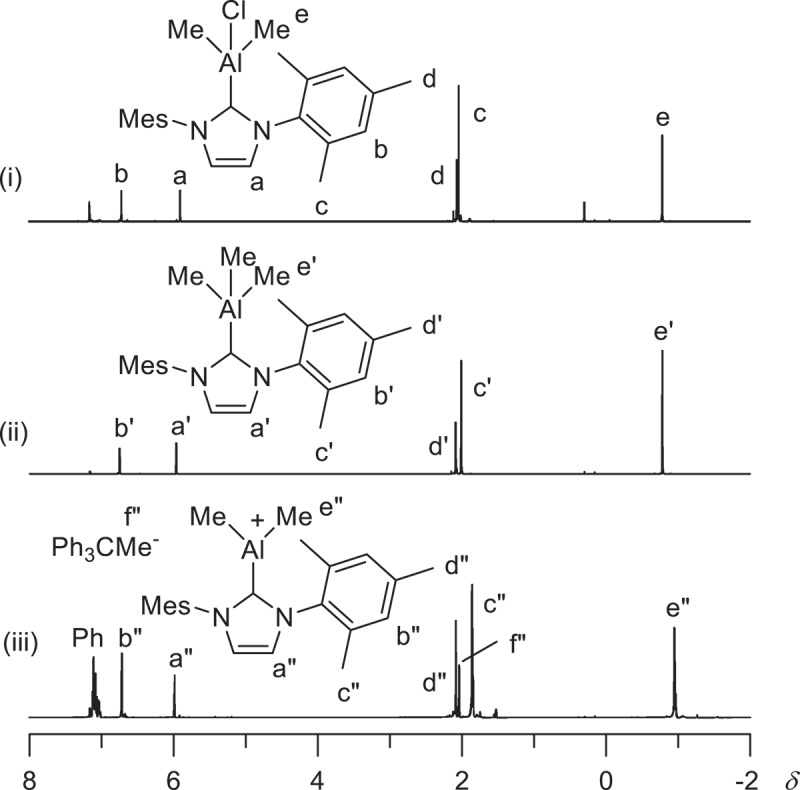
^1^H NMR spectra (in C_6_D_6_ at r.t.) of (i) the product obtained by the reaction of (IMes)AgCl and Me_3_Al, (ii) (IMes)AlMe_3_, and (iii) the reaction mixture between (IMes)AlMe_3_ and Ph_3_C[B(C_6_F_5_)_4_].

Thus, the reaction of less sterically hindered (IMes)AgCl (**5-Ag**) with Me_3_Al was conducted. (IMes)AgCl (**5-Ag**) (0.18 mmol) and Me_3_Al (0.18 mmol) were reacted in toluene (1.8 mL) at r.t. for 12 h. After filtration, the filtrate was concentrated and cooled to −20°C to afford white powder. Figure 4 shows the ^1^H NMR spectra of the produced powder (Figure 4 (i)) and (IMes)AlMe_3_ complex prepared by the reaction of IMes with Me_3_Al according to the procedure reported by Dagorne (Figure 4 (ii)) [39,40].

Chemical shift of the signals due to IMes ligand of the product are close to that of (IMes)AlMe_3_. The signal at δ −0.78 is assigned to AlMe group. Intensity ratio of the signal (e) and that of IMes ligand (b) is 6.0:4.0, which indicates that two methyl group is bonded to Al. Thus, it is speculated that the complex formed by the reaction of (IMes)AgCl and Me_3_Al is (IMes)AlMe_2_Cl (eq. 1). Thus, IMes ligand transfers from silver to aluminum. It has been reported recently that the reaction of (IMes)AgCl and AlCl_3_ affords (IMes)AlCl_3_ via ligand transfer from silver to aluminum [41].

**Figure:**
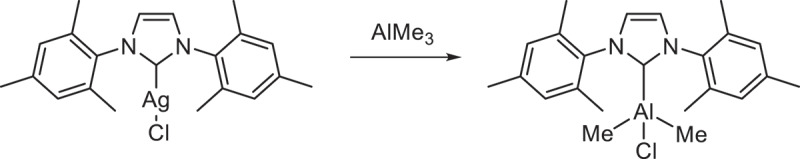

Progress of the ligand transfer reaction was monitored by ^1^H NMR (Figure S2). (IMes)AgCl (**5-Ag**) (0.047 mmol) and Me_3_Al (0.12 mmol, 2 M toluene solution) were mixed in C_6_D_6_ (0.45 mL) and reacted at room temperature (naphthalene (0.025 mmol) was added as internal standard). After the reaction time of 1 h, new signals were observed at δ −0.77 and −0.78 ppm, which are due to (IMes)AlMe_2_Cl formed by the ligand transfer from **5-Ag** to AlMe_3_, and (IMes)AlMe_3_ formed by the reaction between (IMes)AlMe_2_Cl and AlMe_3_, respectively. The relative intensity of the signal at δ 5.94 due to imidazolium group and those due to AlMe is 2.0:3.8. The intensity of the signals due to AlMe becomes larger after 2 h (2.0:6.3) and after 3 h (2.0:11.0). Thus, the ligand transfer reaction from (IMes)AgCl to AlMe_3_ seems to complete in 3 hours, and it is speculated that the reaction is much faster in the presence of large excess of organoaluminum with respect to the silver complex.

Synthesis of the similar NHC aluminum complex by the reaction of silver complex with 1-methyl-3-(pyridylmethyl)imidazolylidene ligand (**1’-Ag**) (0.30 mmol) with Me_3_Al (0.9 mmol, 2 M toluene solution) was conducted similarly to the synthesis of (IMes)AlMe_2_Cl. ^1^H NMR spectrum of the reaction mixture (reaction time = 21 h) showed the signals at δ 8.39, 5.06, and 3.14, which are assigned to vinylene, CH_2_-py, and *N*-Me groups the 1-methyl-3-(pyridylmethyl)imidazolylidene ligand, as well as the signal at δ −0.61, which is assigned to the AlMe group. The intensity ratio of the signal due to CH_2_-py and the signal due to AlMe is 2.0:5.9, which indicates formation of (NHC)AlMe_2_Cl complex (Figure S3). However, minor signals are observed at 7.5 to 2.5 ppm as well as 0.3 to −0.3 ppm, which is due to the presence of other organoaluminum species, such as those with AlMe_3_ coordinated to the pyridyl group. The higher activity of the silver complexes with pyridylmethyl group would be due to the cooperation between the aluminum centers locating on imidazolylidene group and pyridyl group. Attempted isolation of the product by recrystallization was not successful.

Addition of Ph_3_C[B(C_6_F_5_)_4_] (0.025 mmol) to (IMes)AlMe_3_ (0.025 mmol) in C_6_D_6_ (0.5 mL) resulted in upfield shift of the signal due to AlMe from δ −0.79 (e) to δ −0.95 (e”) (Figure 4 (iii)). Relative intensity of the AlMe signal (e, e”) with respect to the signals due to IMes (b, b”) also decreased from 4.0:9.5 to 4.0:7.6. The signal due to Me group abstracted by Ph_3_C^+^ was observed at δ 2.04 (f”). These results indicate the formation of cationic Al complex ([(IMes)AlMe_2_][B(C_6_F_5_)_4_]) via abstraction of a methyl group bonded to Al by Ph_3_C[B(C_6_F_5_)_4_].

## Ethylene polymerization by NHC aluminum complex catalyst

Several aluminum complexes have been known to promote ethylene polymerization in the absence to transition metal complexes [42–47]. Thus, (IMes)AlMe_3_ was examined as transition metal-free catalyst for ethylene polymerization (Table 2). (IMes)AlMe_3_/Ph_3_C[B(C_6_F_5_)_4_] (0.02 mmol/0.02 mmol) showed negligible activity toward ethylene polymerization at 30°C and 50°C (runs 1 and 2). In the presence of ^i^Bu_3_Al (2 mmol), however, (IMes)AlMe_3_/Ph_3_C[B(C_6_F_5_)_4_] afforded polyethylene (run 3). (IMes)AlMe_3_/Ph_3_C[B(C_6_F_5_)_4_]/Me_3_Al showed higher activity than (IMes)AlMe_3_/Ph_3_C[B(C_6_F_5_)_4_]/^i^Bu_3_Al (run 4). ^13^C{^1^H} NMR spectrum of the produced polyethylene indicate linear structure of the polymer without branches (Figure S4). ^i^Bu_3_Al and Me_3_Al are considered to act as scavenger during the polymerization, and prevent deactivation of the cationic aluminum catalyst. The produced polymer showed melting point at 133.3°C, which is lower than those in the silver complex/MAO systems. It indicates lower molecular weight of the polymer by (IMes)AlMe_3_/Ph_3_C[B(C_6_F_5_)_4_]/Me_3_Al. (IMes)AlMe_3_/Ph_3_C[B(C_6_F_5_)_4_]/MMAO is also effective as catalyst, and its activity at 50°C is comparable to (IMes)AlMe_3_/Ph_3_C[B(C_6_F_5_)_4_]/Me_3_Al (runs 5, 6, and 4). Increasing the amount of Ph_3_C[B(C_6_F_5_)_4_] with respect to (IMes)AlMe_3_ resulted in lower activity (run 7).

**Table 2.** Ethylene polymerization by (IMes)AlMe_3_ (**5-AlMe**)/Ph_3_C[B(C_6_F_5_)_4_] or (IMes)AlMe_2_Cl (**5-AlCl**)/NaBARF^.a^

Run	NHC complex	Organoaluminum	Temp. /°C	Yield /g	Activity /g mmol _metal_^−1^ h^−1^	*T*_m_^*d*^ /°C
1	**5-AlMe**	-	30	Trace	-	- ^*e*^
2	**5-AlMe**	-	50	0.0014	0.0035	- ^*e*^
3	**5-AlMe**	^i^Bu_3_Al	50	0.0004	0.0010	- ^*e*^
4	**5-AlMe**	Me_3_Al	50	0.0068	0.0170	133.4
5	**5-AlMe**	MMAO	30	0.0035	0.0088	- ^*e*^
6	**5-AlMe**	MMAO	50	0.0053	0.0130	- ^*e*^
7^*b*^	**5-AlMe**	Me_3_Al	50	0.0033	0.0083	- ^*e*^
8^*c*^	**5-AlCl**	^i^Bu_3_Al	50	0.0013	0.0033	- ^*e*^
9	**5**	MMAO	30	0.0126	0.0315	140.8

^*a*^Reaction Conditions: [NHC complex] = [Ph_3_C[B(C_6_F_5_)_4_]] = 0.020 mmol, [organoaluminum] = 20 mmol, toluene = 20 mL, ethylene = 1MPa, 20 h. ^*b*^[Ph_3_C[B(C_6_F_5_)_4_]] = 0.040 mmol. ^*c*^NaBARF was used instead of Ph_3_C[B(C_6_F_5_)_4_]. ^*c*^Not determined. ^*d*^Determined by DSC, 1st scan. ^*e*^Not determined.

Activity of (IMes)AlMe_2_Cl (**5-AlCl**)/NaBARF/^i^Bu_3_Al was lower than (IMes)AlMe_3_/Ph_3_C[B(C_6_F_5_)_4_]/Me_3_Al (run 8), which would be due to slower formation of cationic aluminum complex in the reaction between (IMes)AlMe_2_Cl and NaBARF (BARF = [B{C_6_H_3_(CF_3_)_2_-3,5}_4_]) in toluene. The IMes ligand (**5**) was also employed in combination with MMAO for the ethylene polymerization, where polyethylene was obtained in higher yield (run 9). The melting point of the produced polyethylene was observed at 140.7°C, which is higher than that obtained by (IMes)AlMe_3_/Ph_3_C[B(C_6_F_5_)_4_]/Me_3_Al (133.3°C). Thus, MAO might play an important role for the production of higher molecular weight polyethylene. Compared to the silver complex/MAO systems, the transition-free Al-based catalyst systems showed lower catalytic activity. Silver is considered to enhance activity of the Al catalyst, but its exact role is not clear at present.

## Conclusion

NHC silver complexes promote ethylene polymerization in the presence of MMAO. The produced polyethylene is rarely soluble in common organic solvent, but DSC analysis and SEM observation indicate ultra-high molecular weight of the produced polyethylene. Investigation of the reaction between the NHC silver complex with organoaluminum indicates the NHC ligand transfer from silver to aluminum takes place. Thus, the NHC aluminum complex is considered to be the actual active species of the polymerization. The present catalyst must be also valuable from a viewpoint of transition-metal-free olefin polymerization catalyst. However, the use of NHC silver complexes has advantage to easily introduce less stable NHC ligand to aluminum center.

## Experimental section

### General method

All manipulations of air- and water-sensitive compounds were carried out under nitrogen. NMR spectra were recorded on JEOL JNM-ECZ500R spectrometer at 20°C (in C_6_D_6_) or 130°C (in C_2_D_2_Cl_4_). ^1^H (500 MHz) and ^13^C{^1^H}(125 MHz) NMR chemical shifts were referenced to C_6_H_6_ (δ 7.16) in the C_6_D_6_ solvent or C_2_H_2_Cl_4_ (δ 5.91) in the C_2_D_2_Cl_4_ solvent for ^1^H and C_6_D_6_ (δ 128) or C_2_D_2_Cl_4_ (δ 74.2) for ^13^C. High temperature GPC A morphology of the polyethylene was observed as a secondary electron image using a Hitachi S-4800 field-emission scanning electron microscope (SEM) operated at an accelerating voltage of 1.0 kV and a working distance of 8.4 mm. The sample was coated with Pt – Pd before the observation. DSC was recorded on Seiko DSC6200R instruments (1^st^ heating and cooling, heating rate = 10°C/min, cooling rate = −20°C/min).

### Materials

Dry solvents were purchased and used as received unless otherwise noted. C_6_D_6_, distilled from Na/benzophenone, was used for NMR measurement. Organoaluminum compounds (MAO, MMAO, MMAO-12, DEAC, ^i^Bu_3_Al, Me_3_Al) were purchased and used as received. NHC ligands and complexes **1-Ag** [48], **1’-Ag** [49,50], **2-Ag** [48], **3-Ag**, [51] **4-Ag** [52], **5-Ag** [53], and **5’-Ag** [52] were synthesized according to the literature method.

### Reaction of NHC or NHC silver complex with organoaluminum

NHC or NHC silver complex was placed in 50 mL Schlenk flask. The reaction vessel was dried in vacuo, and N_2_ gas was purged. Toluene was added to the flask, and the mixture was stirred overnight at room temperature. The reaction mixture was filtered through celite and filtrate was concentrated under reduced pressure. The solution was recrystallized at −20℃ to afford the crystal of NHC aluminum complexes.

### Polymerization reactions

NHC silver complex was placed in 100-mL autoclave and was dried in vacuo. The reaction vessel was purged with 1 MPa ethylene and degassed. This process was repeat three times. And then, toluene was added to the reaction vessel and 1 MPa ethylene was purged. After stirred at 750 rpm for 20 h, the reaction mixture was poured to HCl/MeOH, and formed white precipitate was collected by filtration.

## Supplementary Material

Supplemental Material

## Data Availability

The data that support the findings of this study (^1^H NMR spectra and DSC charts) are available in the supporting information https://doi.org/10.6084/m9.figshare.23585295.
