# Supplementary material for: Ethylene polymerization using N-Heterocyclic carbene complexes of silver and aluminum
Source: Des Monomers Polym. 2023 Jul 5;26(1):182–9. doi: 10.1080/15685551.2023.2229641 (PMC10324440; doi:10.1080/15685551.2023.2229641)
Supplement: Supplemental Material [file TDMP_A_2229641_SM5847.pdf]

Electronic Supporting Information for

Ethylene Polymerization Using *N*-Heterocyclic Carbene Complexes of Silver and Aluminum

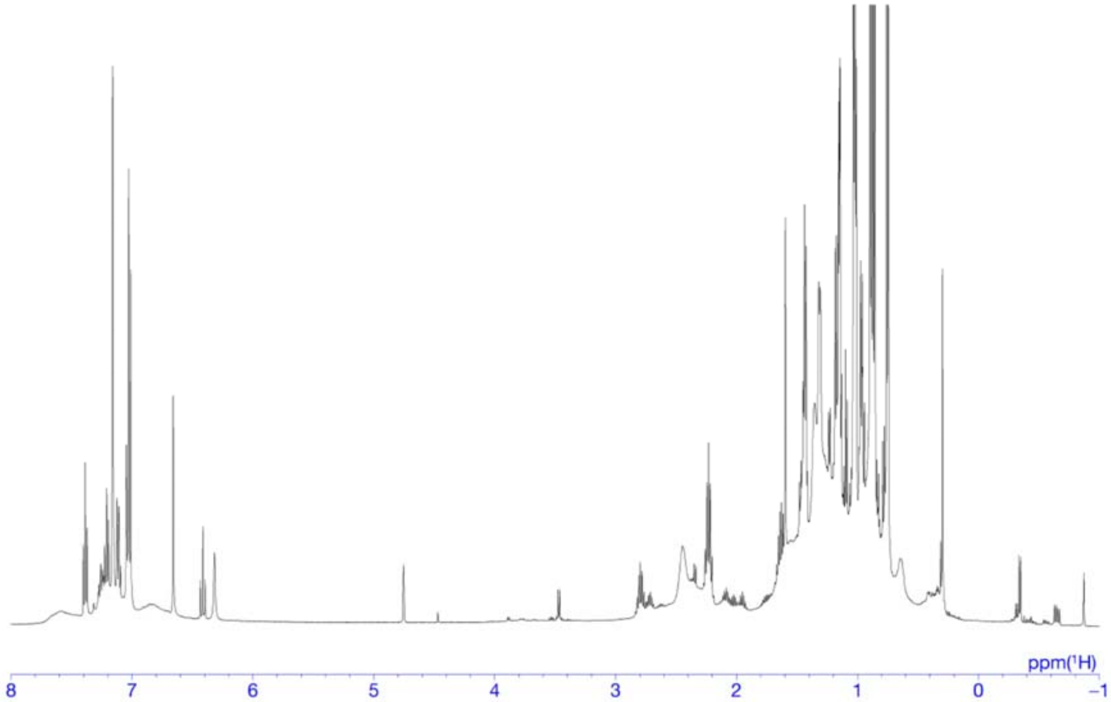

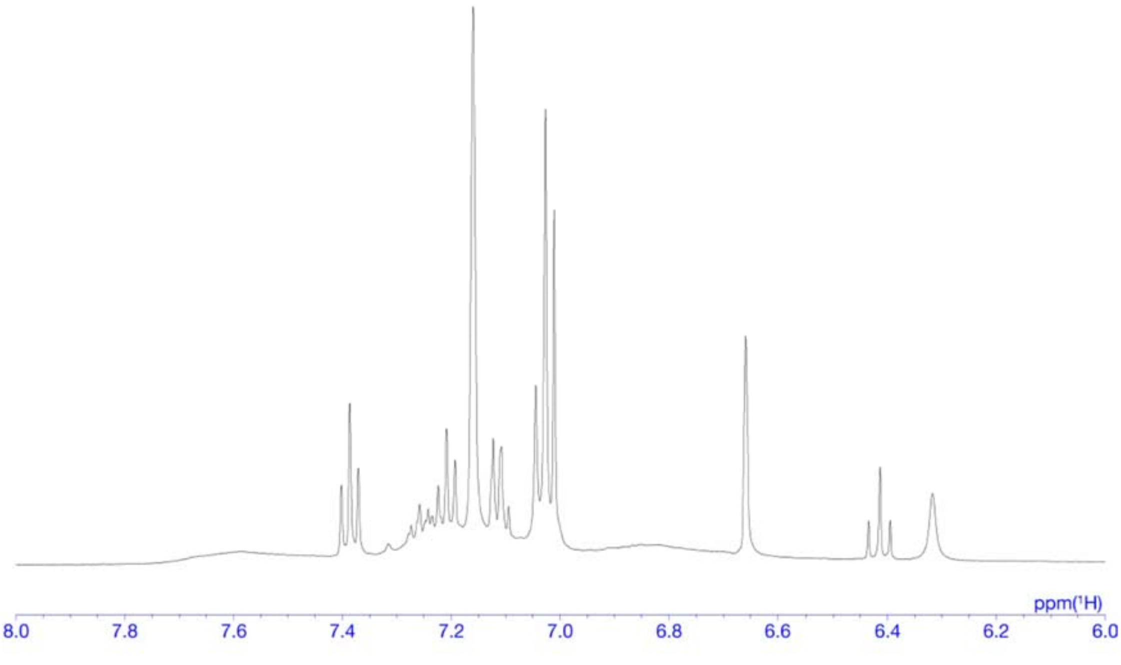

Figure S1. ^{1}H NMR spectra of the reaction mixture of **6-Ag** and $^{i}Bu_3Al$ in C_6D_6 measured at 24 h after the preparation of the reaction mixture.

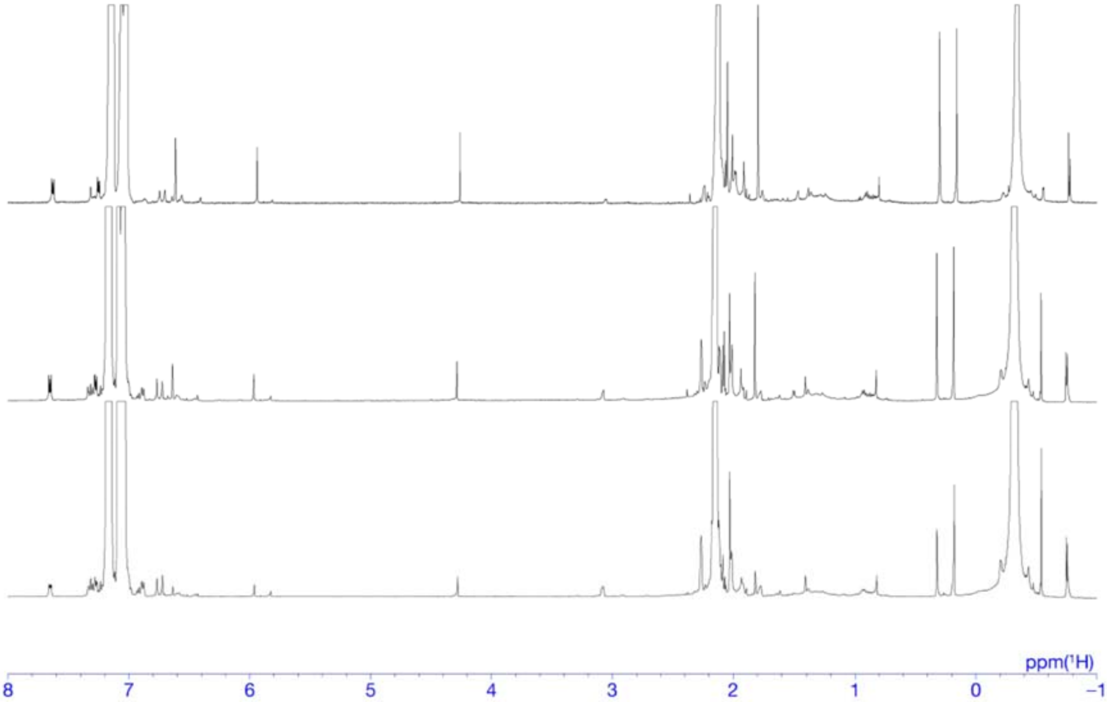

Figure S2. ¹H NMR spectra of the reaction mixture of **5-Ag** and Me_3Al in C_6D_6 measured at 1 h (top), 2 h (middle), and 3 h (bottom) after the preparation of the reaction mixture.

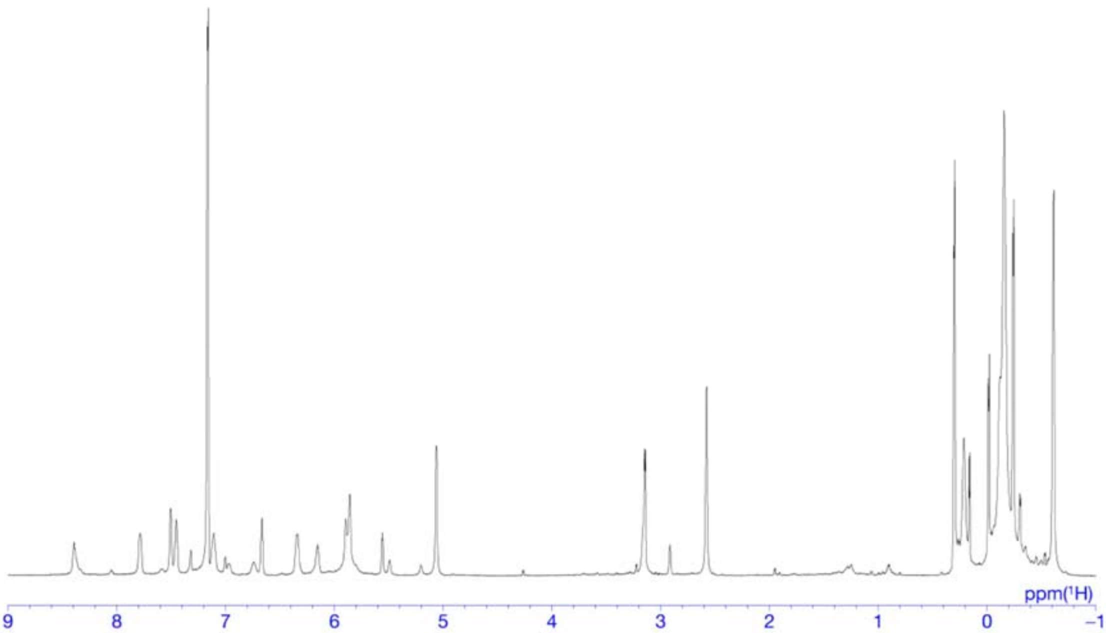

Figure S3. ^{1}H NMR spectra of the reaction mixture of **1’-Ag** and Me_3Al in C_6D_6.

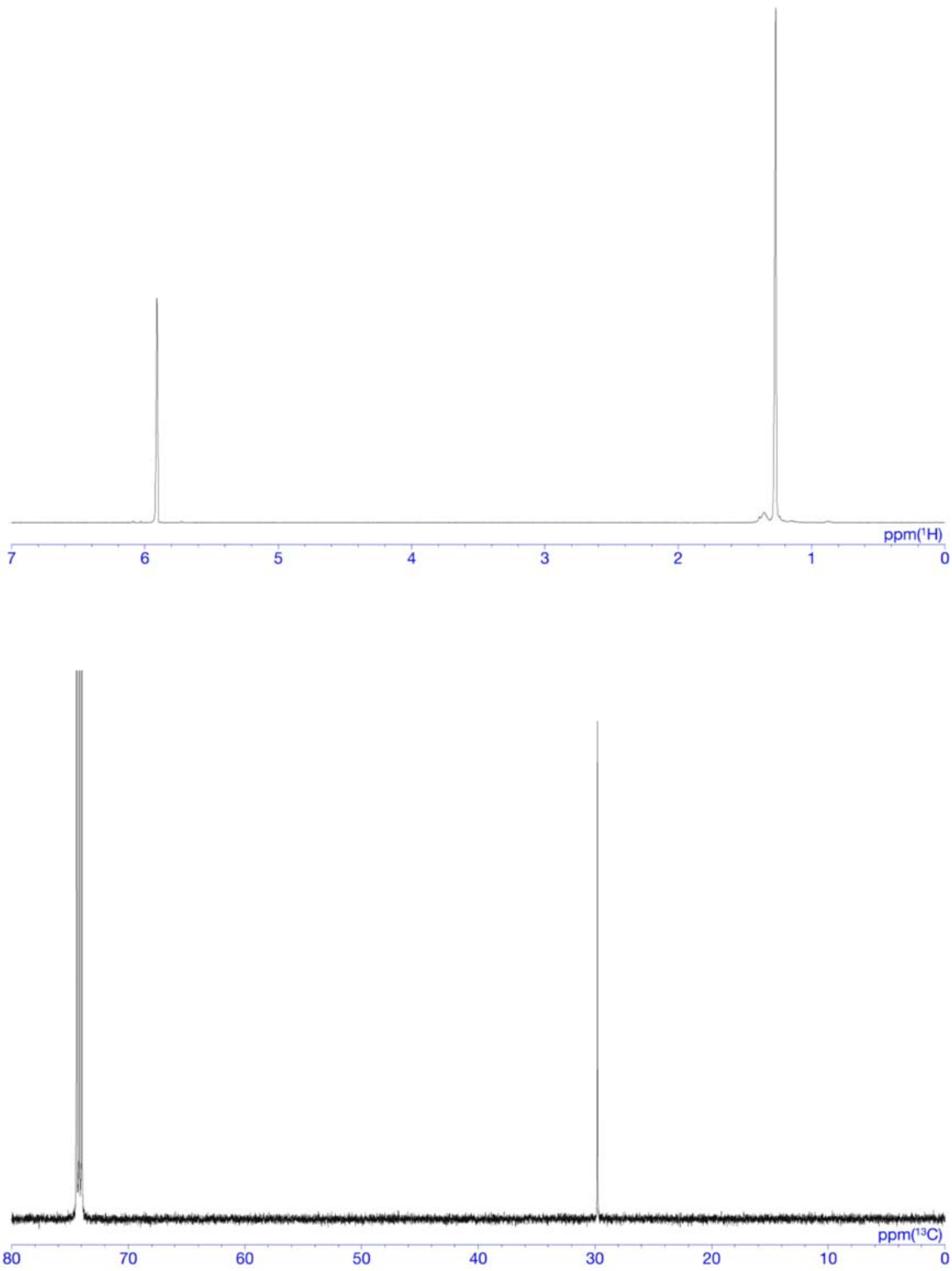

Figure S4. ^{1}H (top) and $^{13}C\{^{1}H\}$ NMR spectra ($C_2D_2Cl_4$, 130 °C) of polyethylene obtained by (IMes)$AlMe_3$/$Ph_3C[B(C_6F_5)_4]$/$^{i}Bu_3Al$ (Table 2, run 4).

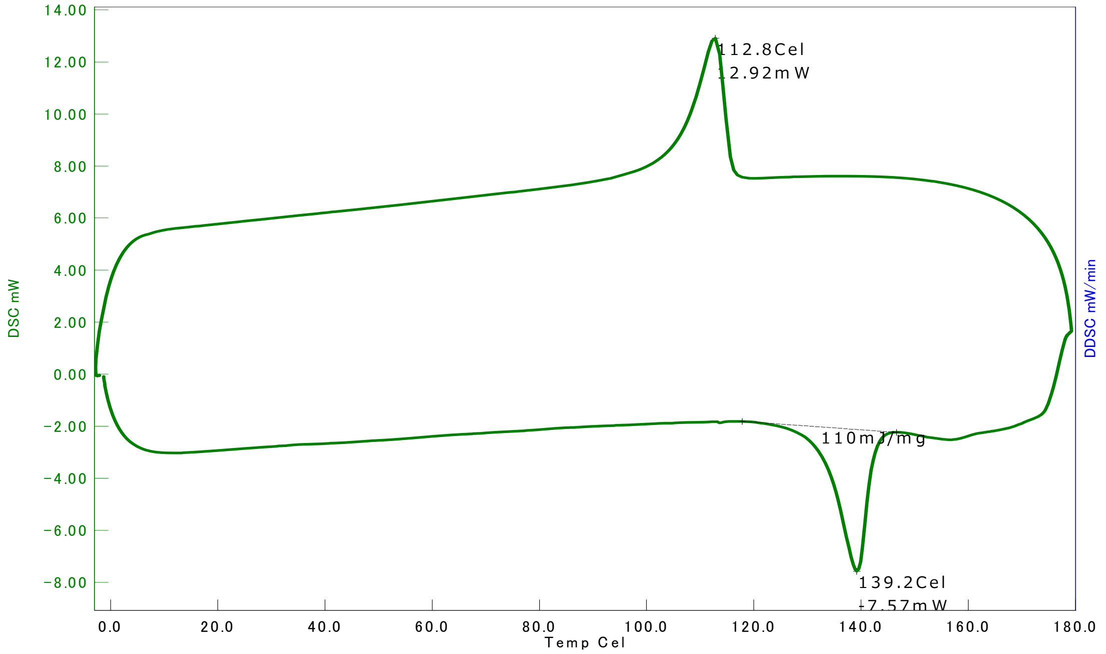

Figure S5. DSC profile of polyethylene (Table 1, run 1, 1st heating and cooling)

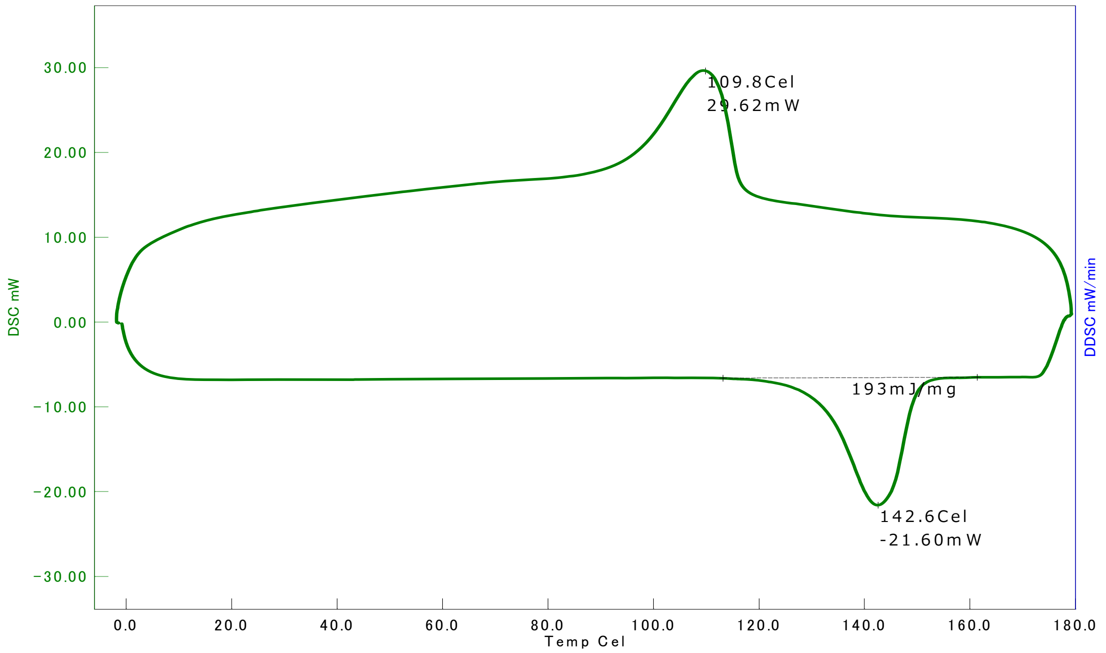

Figure S6. DSC profile of polyethylene (Table 1, run 2, 1st heating and cooling)

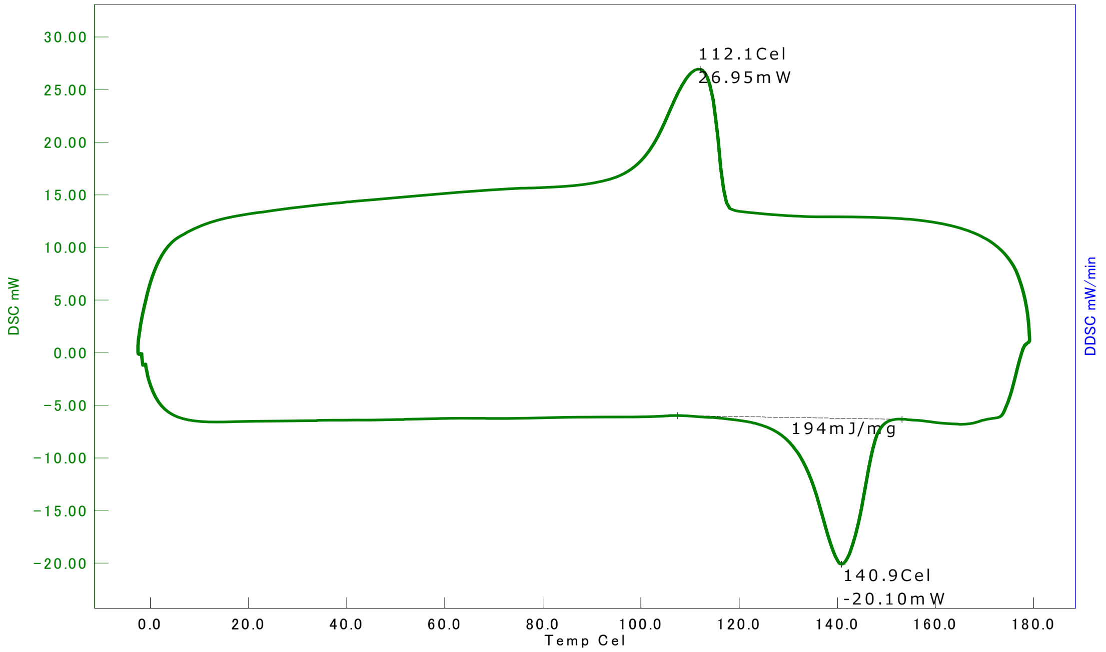

Figure S7. DSC profile of polyethylene (Table 1, run 6, 1st heating and cooling)

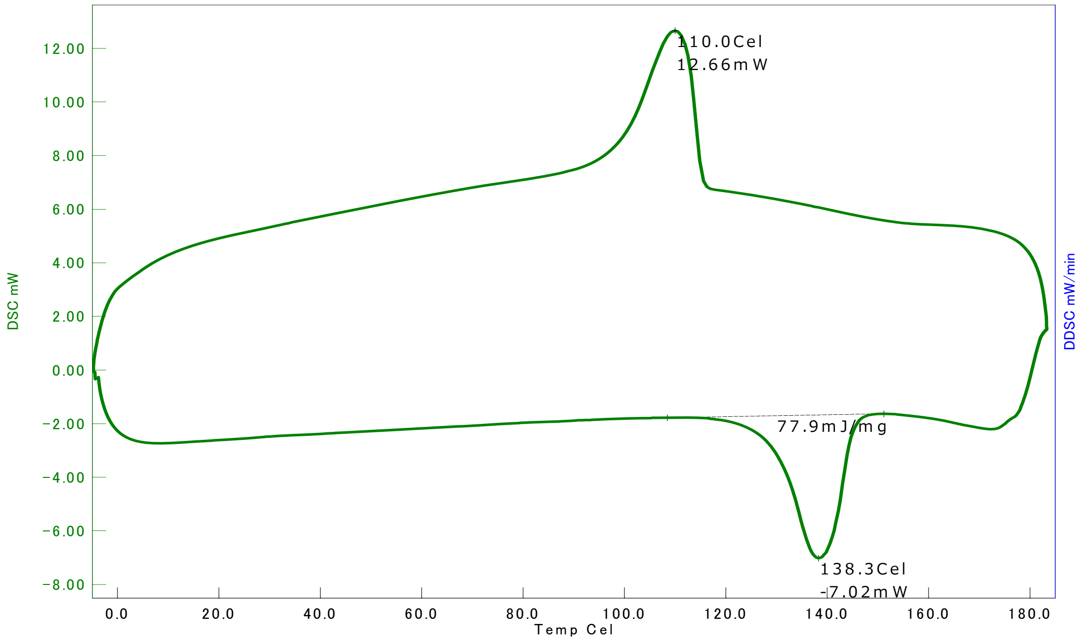

Figure S8. DSC profile of polyethylene (Table 1, run 7, 1st heating and cooling)

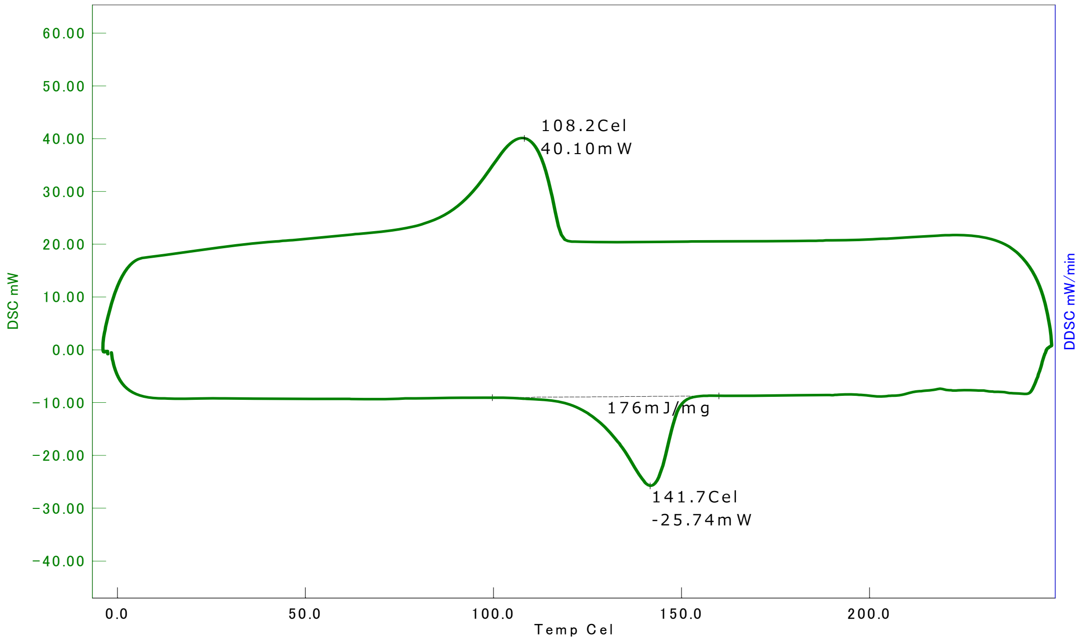

Figure S9. DSC profile of polyethylene (Table 1, run 8, 1st heating and cooling)

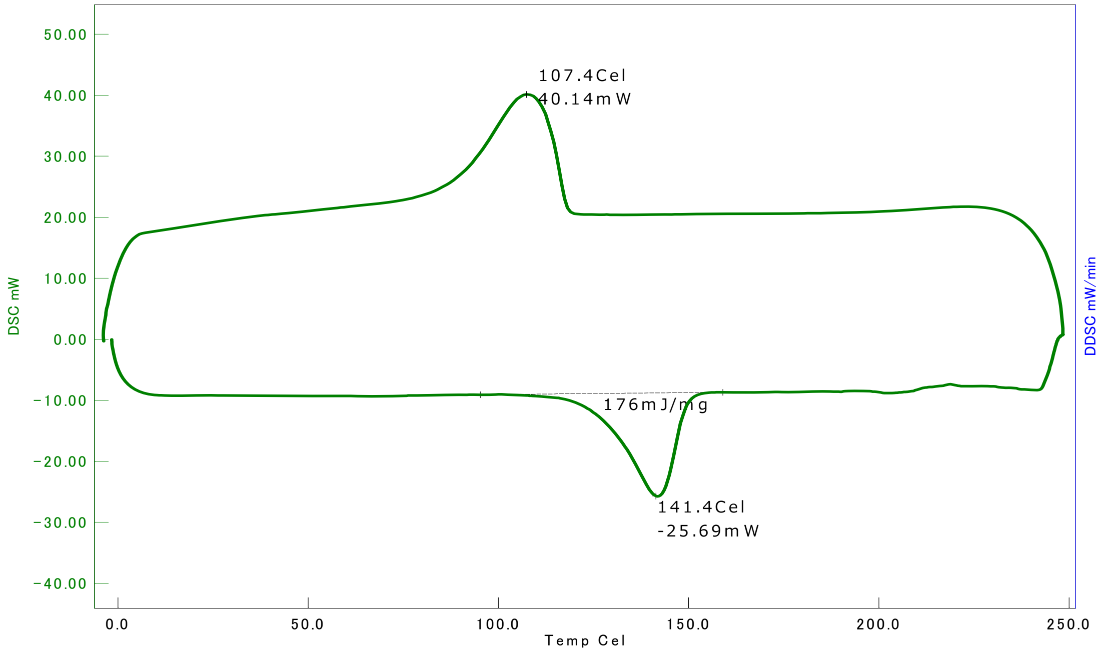

Figure S10. DSC profile of polyethylene (Table 1, run 9, 1st heating and cooling)

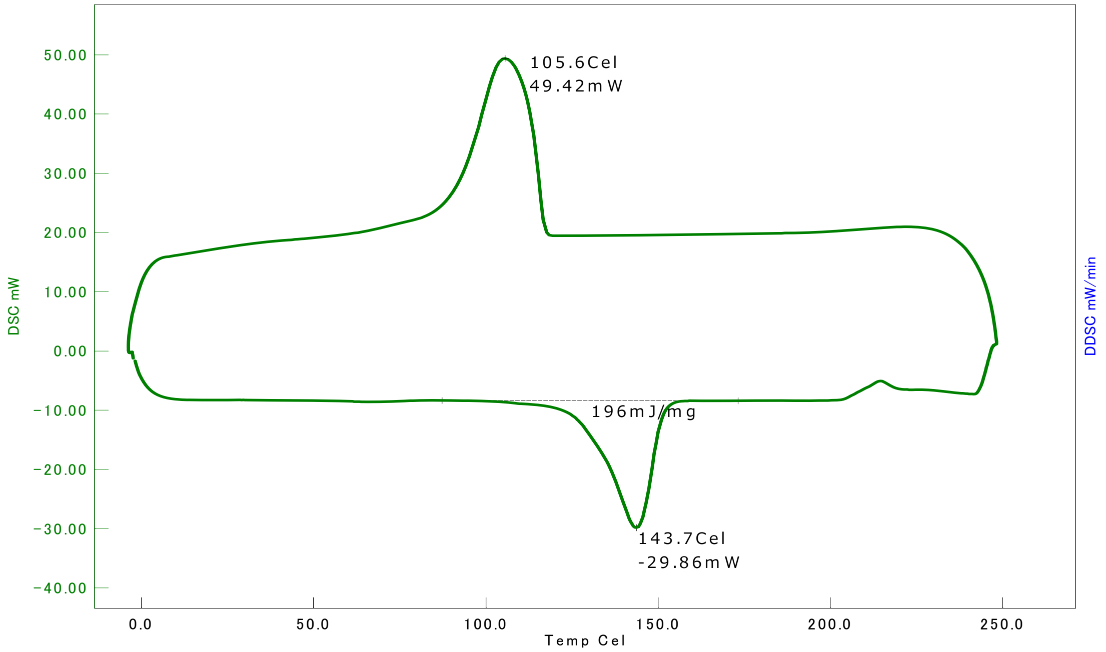

Figure S11. DSC profile of polyethylene (Table 1, run 12, 1st heating and cooling)

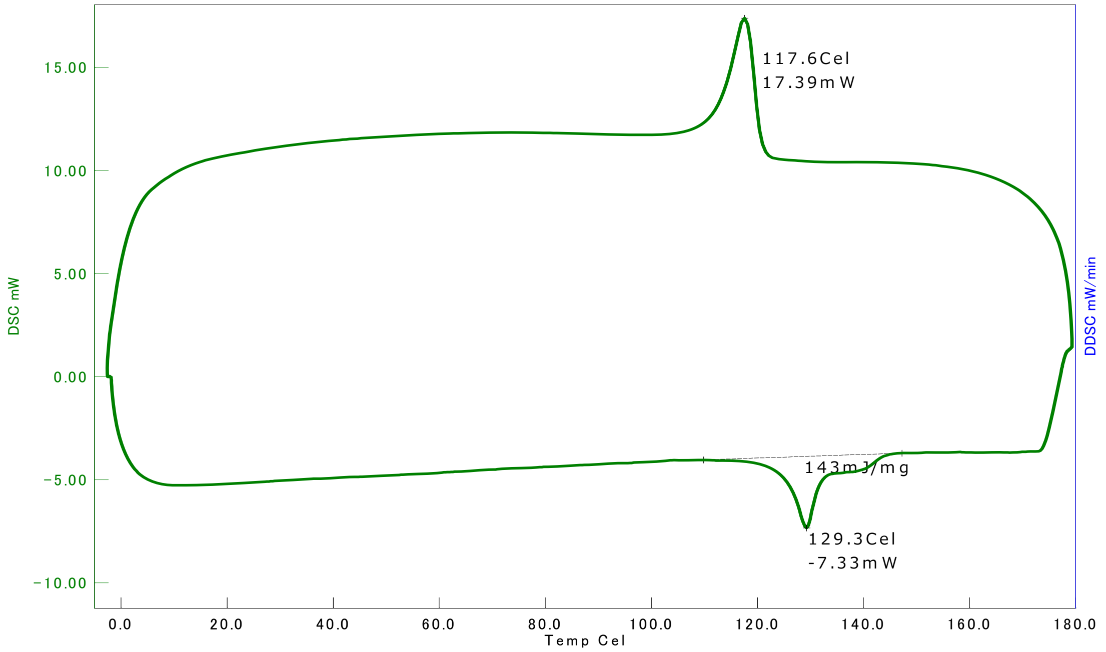

Figure S12. DSC profile of polyethylene (Table 1, run 15, 1st heating and cooling)

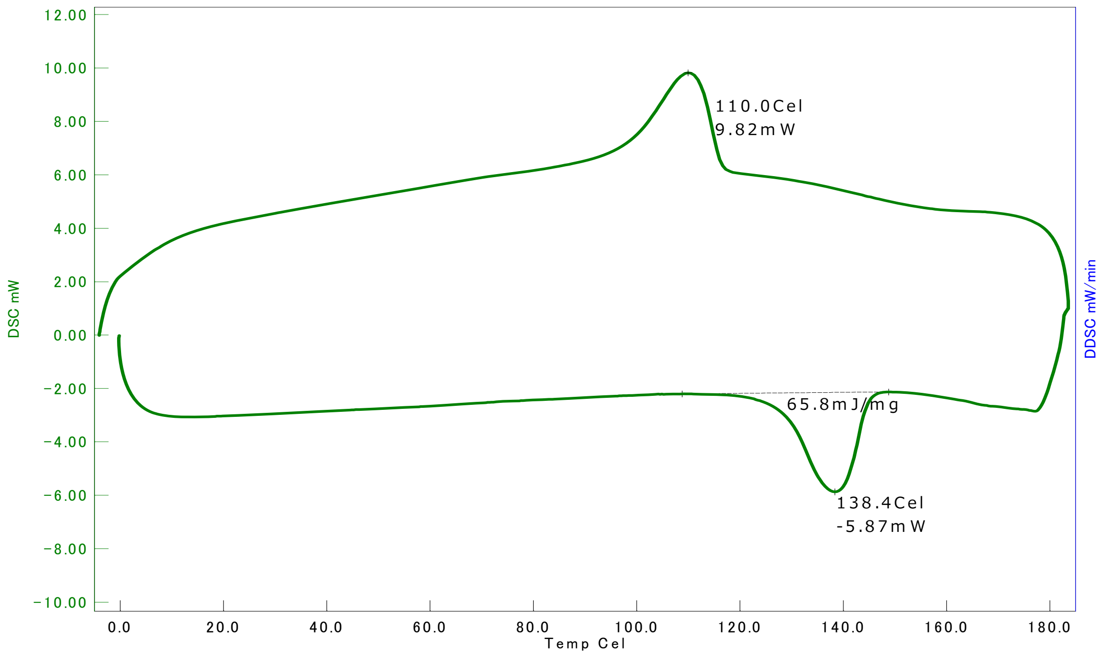

Figure S13. DSC profile of polyethylene (Table 1, run 16, 1st heating and cooling)

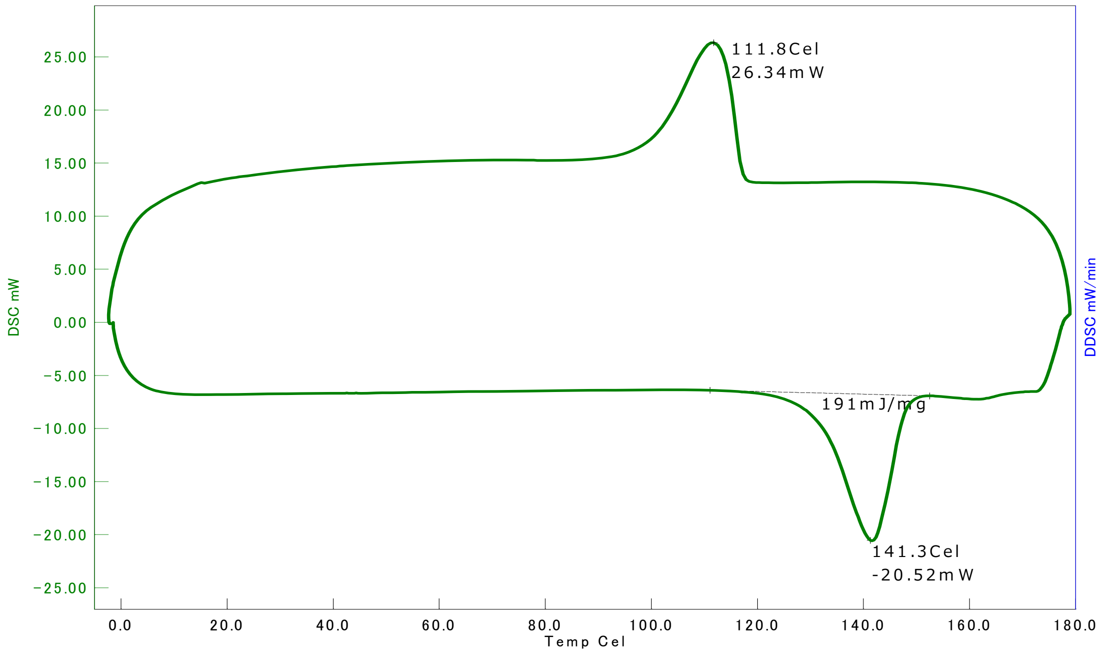

Figure S14. DSC profile of polyethylene (Table 1, run 17, 1st heating and cooling)

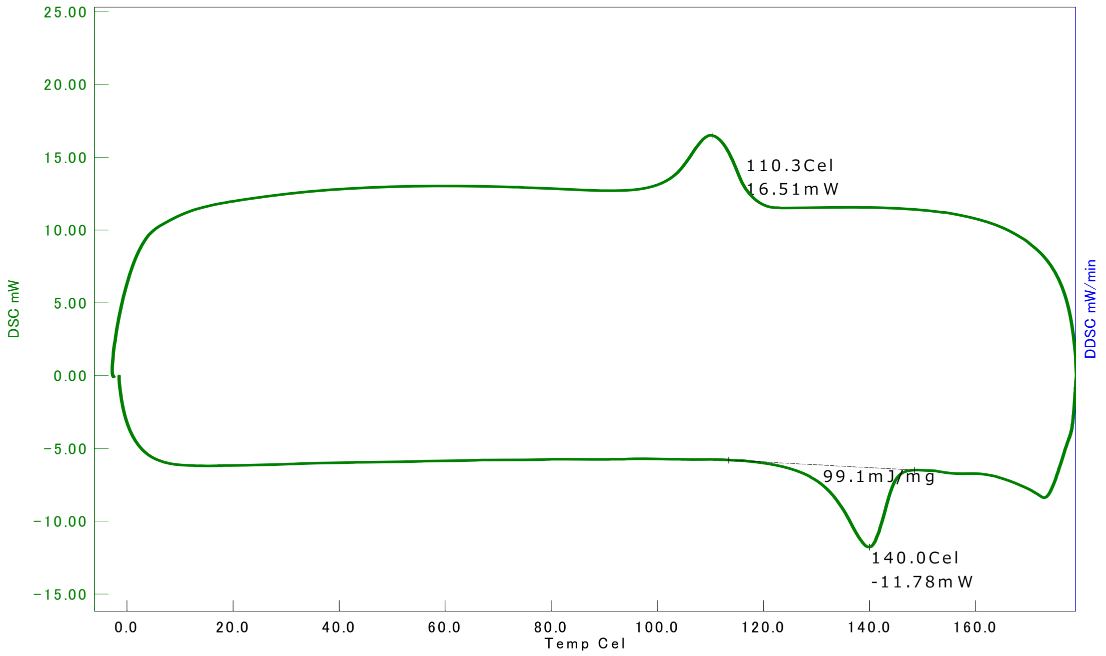

Figure S15. DSC profile of polyethylene (Table 1, run 18, 1st heating and cooling)

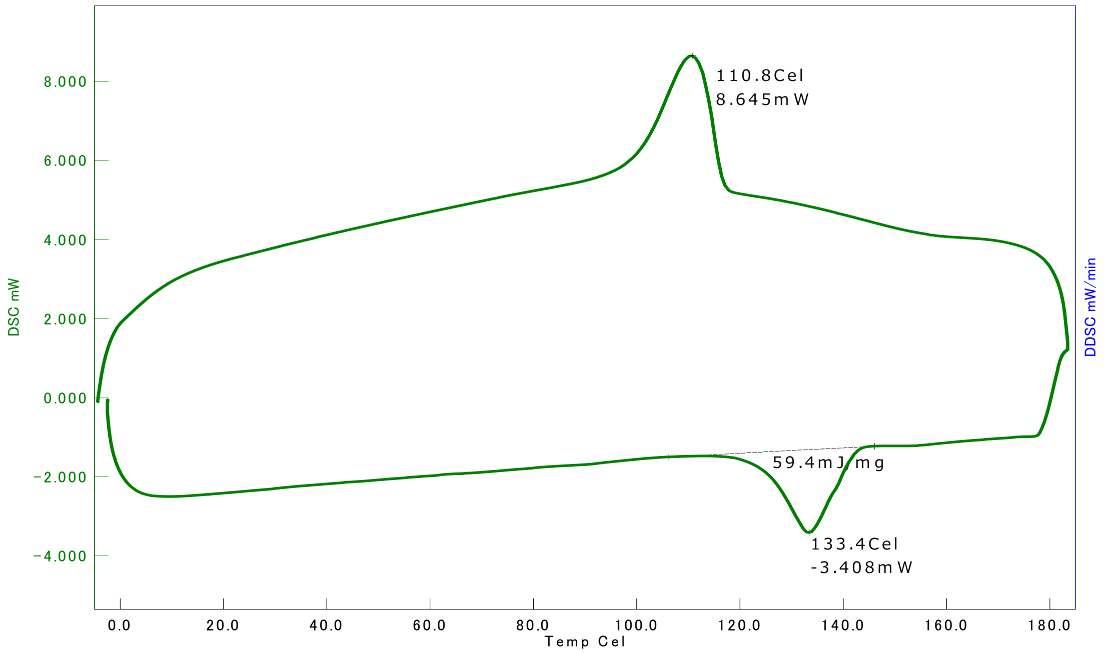

Figure S16. DSC profile of polyethylene (Table 2, run 4, 1st heating and cooling)

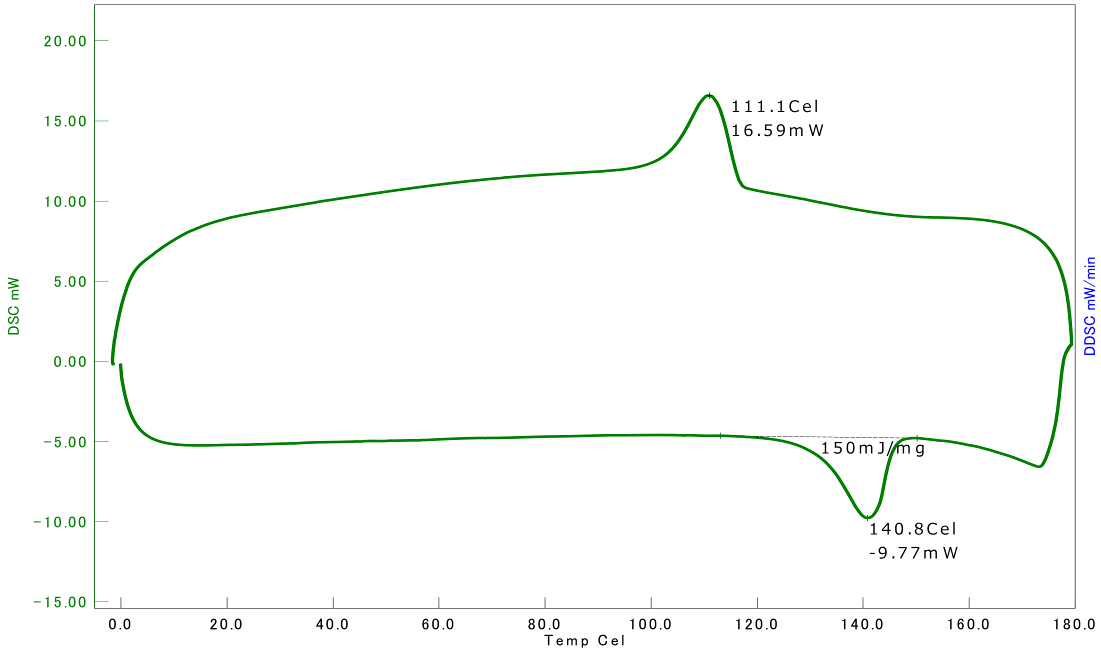

Figure S17. DSC profile of polyethylene (Table 2, run 9, 1st heating and cooling)
